# An Early Intestinal Cancer Prediction Algorithm Based on Deep Belief Network

**DOI:** 10.1038/s41598-019-54031-2

**Published:** 2019-11-22

**Authors:** Jing-Jing Wan, Bo-Lun Chen, Yi-Xiu Kong, Xing-Gang Ma, Yong-Tao Yu

**Affiliations:** 1 Department of Gastroenterology, The Affiliated Huai’an Hospital of Xuzhou Medical University, the Second People’s Hospital of Huai’an, Huaian, 223002 China; 2 College of Computer Engineering, Huaiyin Institute of Technology, Huaian, 223003 China

**Keywords:** Information technology, Information theory and computation, Gastrointestinal models

## Abstract

The incidence of colorectal cancer (colorectal cancer, CRC) in China has increased in recent years, and its mortality rate has become one of the highest among all cancers. CRC also increasingly affects people’s health and quality of life, and the workloads of medical doctors have further increased due to the lack of sufficient medical resources in China. The goal of this study was to construct an automated expert system using a deep learning technique to predict the probability of early stage CRC based on the patient’s case report and the patient’s attributes. Compared with previous prediction methods, which are either based on sophisticated examinations or have high computational complexity, this method is shown to provide valuable information such as suggesting potentially important early signs to assist in early diagnosis, early treatment and prevention of CRC, hence helping medical doctors reduce the workloads of endoscopies and other treatments.

## Introduction

CRC is a common malignant tumor in China. As people’s living standards have continued to improve and changes in people’s eating habits, the incidence and mortality of CRC have continued to rise, seriously endangering the health and quality of life of the Chinese people. According to Chinese cancer statistics from 2015, the incidence and mortality of CRC ranked fifth among all malignant tumors, including nearly 400,000 new cases and nearly 200,000 deaths, a mortality of 50%^1^. In addition, a recently published study showed a significant increase in the annual rate of CRC incidence among young people^2^. Due to its high morbidity and mortality, CRC prevention is an urgent problem that needs to be addressed.

CRC prognosis is closely related to its early diagnosis. Most CRC cases can be cured when they are discovered at an early stage; the 5-year survival rate after early diagnosis can be as high as 90%. In contrast, when discovered only in the later stages, the 5-year survival rate is less than 10%^3^. In the clinic, early diagnosis and early treatment are generally conducted by screening to reduce the incidence and mortality of CRC. Colonoscopy is the primary means of early diagnosis. However, domestic and foreign studies have shown that CRC screening programs for early diagnosis are not sufficiently accurate; only a small number of cases are screened out among a large number of people, resulting in low screening compliance among patients^4,5^.

In addition, in China, the heavy workloads of medical professionals are well known^6^, and a series of social and economic problems have been reported^7–10^. These problems are mainly due to the insufficiency of medical resources in China and the inefficient allocation of medical resources. Moreover, such causes will likely be difficult to address in the short term. Therefore, we believe that a technical approach can partially reduce doctors’ workloads—that is, by freeing doctors from repetitive work that does not require in-depth thinking. The goal of this study is to reduce doctors’ workloads by designing an automated forecasting system to assist them to make decisions more easily.

Previous early CRC predictions were conducted on a case-by-case basis, using either statistical analyses or patient records. However, a generalized predictive mechanism has yet to be developed because we do not yet fully understand the mechanism of CRC^11^. Thus, a solution to the prediction problem has great practical value. For example, biological field research has linked the protein interaction network and the metabolic network node through an interaction relationship. Revealing the hidden interactions in such networks has high experimental costs; however, the results of the prediction methods can guide experiments and increase their success rates, thereby reducing their costs. Studying disease-gene network losses and predicting suspicious links aids in exploring the mechanism behind the disease, in predicting and evaluating corresponding treatments, and finding new drug targets, thereby opening up new avenues for drug research and development^12^.

The medical industry has incorporated high tech solutions such as artificial intelligence and sensing technologies, making medical services increasingly intelligent. The recent policy of “New Healthcare Reform” in China has made intelligent healthcare care accessible to ordinary people. Intelligent healthcare aims to capitalize on artificial intelligence technology to assist in various types of medical decision making, including disease risk prediction, intelligent healthcare consultation, medical image analysis, electronic medical record information extraction, medical health data analysis, medical insurance evaluation, and making recommendations for medication. In 2017, Esteva developed a deep neural network that can successfully classify skin cancer from sample data^13^, demonstrating that deep learning methods have great potential for use in medical fields. Intelligent systems that can make early disease predictions or help provide information for doctors during the diagnosis process are valuable in both scientific research and clinical medicine.

In recent years, many research teams have attempted to pursue machine learning methods to classify cancer patients as high or low risk. These technologies can play important roles in research and treatment of cancer diseases^14^. The purpose of machine learning methods is to detect key features from complex sample data and to reveal their contributions. Machine learning methods such as artificial neural networks, Bayesian networks, support vector machines (SVM), and decision trees have been widely used in cancer research and provide effective and accurate basic models for early prediction of various types of cancers.

The dimensions of the sample data increase with the number of examination data items during the early diagnosis of cancer. However, because the specific examination items collected vary on a case-by-case basis, it is natural to see data sparseness in the constructed sample dataset. Consequently, the noise in the data also increases, which inevitably negatively impacts the performances of early CRC prediction algorithms. In addition, because of the high dimensionality of the sample data, the time complexity of traditional prediction algorithms is usually high. Therefore, we intend to devise a method to effectively address both data sparsity and high dimensionality and to eliminate noise in prediction problems, allowing us to learn which sample features play key roles in early CRC prediction.

Wang *et al*. defined the problem of feature selection as a combinatorial optimization or search problem in intelligent healthcare, rather than the commonly used filtering, packaging and embedded feature selection methods^15^. They applied several feature selection methods, including exhaustive search, heuristic search and hybrid methods. The heuristic search methods include feature ordering metrics either with or without data extraction. Kleftogiannis *et al*. combined an SVM with a genetic algorithm (GA) to perform feature selection and parameter optimization^16^. Duan proposed a backward elimination feature extraction method similar to the SVM recursive feature elimination method (SVM-RFE)^17^. The method classifies the feature ranking scores by statistically analyzing the weight vectors of the plurality of linear SVMs trained on subsamples of the original training data at each step. Zhong *et al*. used an SVM to analyze protein characteristics based on the Pearson correlation coefficient to eliminate redundant features^18^. Fong *et al*. combined the particle swarm optimization algorithm with three different classification methods—pattern network, decision tree and naive Bayes—to search for the optimal feature subset^19^. The results show that the method achieves high classification precision on specific datasets. Inspired by evolutionary algorithms, Mohapatra *et al*. proposed a modified cat swarm optimization (MCSO) algorithm to extract features from datasets, applied it to several biomedical datasets, and achieved favorable results^20^. Metsis *et al*. proposed a feature extraction method based on a structural sparse induction specification and compared it with existing feature extraction methods on four published ACGH datasets^21^. Boreto *et al*. proposed an analytical geometric feature extraction method to supervise variational correlation learning (suvrel) using a variational method that determines the tensor of the metric to define the distance-based similarity during pattern classification^22^. The variational method was applied to a cost function that penalizes the distance within the large class and the distance within the preferred class. Their approach yields a metric tensor that minimizes the cost function. Bennasar *et al*. introduced the joint mutual information maximization (JMIM) and the normalized joint mutual information maximization (NJMIM) methods, both of which use the maximum value of mutual information and minimum criteria, thus alleviating the theoretical and experimental overestimation of the meanings of features^23^. Xu *et al*. used the minimum redundancy maximum correlation (MRMR) metric, forward feature extraction and an SVM, and found that this combination outperformed other classifiers such as Bayesian decision theory, *K* nearest neighbor and random forest^24^.

In addition, to address the sparsity and noise of the data in such problems, the matrix decomposition technique is a commonly used method at present; its implementation is relatively simple and its prediction accuracy is relatively high. The most famous matrix decomposition methods include singular value decomposition (SVD)^25,26^, principal component analysis (PCA)^27^, independent component analysis (ICA)^28^, and others. Among these, SVD requires completing the data to avoid the sample sparseness problem; however, this operation not only increases the required data storage space but also potentially violates the practical significance of the sample data in a specific environment. Meanwhile, because SVD is a highly complex algorithm, it is not applicable to networks with large sample sizes. Therefore, based on SVD, Simon Funk proposed the LFM model by optimizing the diagonal array of the eigenvalues of the sample data matrix into a decomposed matrix by optimizing the evaluation index RMSE in the training matrix^29^. In real prediction systems, no uniform standard exists for each new data sample; therefore, Koren added the user’s historical scores based on LFM and proposed the SVD++ model^30^.

However, the above series of feature extraction models do not consider the existence of negative values in the sample data. In a prediction system, negative values in the sample matrix have no practical meaning in a real situation. For example, during early cancer diagnosis, a certain patient attribute or a certain indicator with a negative value may be meaningless when reconstructing the sample data. Therefore, Lee and Seung proposed a nonnegative matrix factorization method (NMF)^31,32^, which finds the low rank of the matrix and then decomposes it into a nonnegative matrix. This method not only greatly reduces the dimensionality of the matrix but also removes redundant data, making the decomposed result more interpretable in practice. NMF technology has been widely applied in the health care^33^, medical imaging^34–36^ and biomedical fields^37,38^; however, this technology has not attracted widespread attention in early cancer prediction. Therefore, this paper integrates NMF and combines it with a deep learning method to facilitate early CRC detection.

Multiple examples of deep learning applications exist in medical research, most of which focus on automatically identifying tumor images or detecting gene sequences, and these algorithms have achieved good results. Xiao *et al*. developed a deep learning-based 5-class model to make cancer predictions using RNA sequence data^39^. Danaee *et al*. used a deep learning approach (a stacked denoising autoencoder) to analyze gene expression data and identify genes potentially correlated with breast cancer^40^. Some researchers have applied deep learning techniques to analyze cancer imagery. Bychkov *et al*. proposed a deep learning method to analyze CRC images, and their results showed that state-of-the-art deep learning techniques are able to extract more prognostic information from the tissue morphology of CRC than can an experienced medical professional^41^. Cruz-Roa *et al*. presented and evaluated a deep learning model for automated basal cell carcinoma cancer detection that learns the image representation, performs image classification, and interprets the results^42^. Coudray *et al*. discovered that a deep learning method can classify and predict the mutation of non–small cell lung cancer from histopathology images^43^. Other researchers have also employed deep learning methods to investigate other types of medical data related to cancer prediction. Mamoshina *et al*. used deep neural networks (DNNs) to analyze ‘omics data and achieved state-of-the-art results^44^. Burke *et al*. used artificial neural networks to analyze the American College of Surgeons’ Patient Care Evaluation (PCE) data and obtained improved predictions of patient 5-year survival rates^45^.

However, in real conditions, especially those in developing countries, examination data such as tumor imagery and genetic testing data are not easily obtained. Given the constraints on patients’ economic and medical conditions, numerous patients do not have access to these techniques. In addition, test procedures such as tumor imaging and genetic testing are typically performed only for patients already strongly suspected of having cancer. Therefore, during the most important period (i.e., the prevention and early diagnosis period), these data provide minimal help. In this paper, we attempt to use the simplest and most commonly available test data—the medical examination report—to create a new prediction system to help doctors make decisions. The medical examination report is a basic test that almost every patient undergoes; thus, our early cancer prediction system can be applied to a broader range of patients.

CRC is a multifactor disease. In CRC prediction, combining data such as age, gender, family history of CRC, BMI, past history and other attributes and patient case reports using deep learning techniques in an expert system to predict the likelihood of early cancer will greatly reduce missed diagnoses by clinicians during endoscopy and treatment and will also provide effective help for early diagnosis, early treatment and prevention of CRC.

This paper explores and analyzes patient data from a deep learning perspective combined with patient attributes and case reports to construct an expert system to predict the probability of early cancer. Due to its relatively effective dimensional reduction and noise cancellation techniques, this method shows great promise for application in real scenarios. By greatly reducing missed clinician diagnoses during endoscopy and treatment, it will provide effective help for the early diagnosis, early treatment and prevention of CRC.

## Results

The sample dataset includes each sample’s attributes (e.g., age, gender, smoking history, and drinking history), endoscopic features (e.g., lesion location, polyp size, and no leaf) and blood attributes (e.g., white blood cells and hemoglobin). There are 50 features in all categories.

We compare early cancer prediction (ECP) using four classic machine learning algorithms, i.e., an (SVM), KNN, ensembles for boosting (EB), and random forest (RF), and three deep learning methods, i.e., a CNN, a recurrent neural network (RNN1), and a recursive neural network (RNN2). Each method’s performance is averaged over 100 runs in which the data are randomly separated into a training set (containing 90% of the links) and a test set (including 10% of the links). Normally, precision and recall are not necessarily related; however, in large-scale datasets, these two indicators are correlated. A false negative example (FN) means that the prediction model incorrectly predicted a sample from the positive category as a negative category. Specifically, in this experiment, a FN means that a sample from a cancer patient was classified as being from a noncancer patient. In the clinic, the false negative rate (FNR) is important because it may lead to a missed diagnosis. Therefore, in this paper, we mainly use the F1_Score and FNR as the evaluation metrics of the algorithms. The experimental results are as follows:

From Table 1, we can see that our ECP algorithm achieves the highest F1_Score on the real sample dataset. Both the Precision and Recall of our method outperform other algorithms. In addition, the FNR is the smallest among all algorithms. After dimensional reduction by a nonnegative matrix, we reduced the original 50-dimensional matrix to 14 dimension and extracted the hidden features. This idea facilitates effective early diagnosis, early treatment and prevention of cancer. Therefore, our algorithm not only reduces the spatial complexity of the sample but also achieves better prediction results. False negatives can also be caused by instability in the patient’s condition, and related data may be collected during the window period of other diseases, resulting in data noise.

**Table 1:** Comparison of the prediction results of five algorithms measured by different evaluation indices,the best results are marked with bold.

Networks	Accuracy	Precision	Recall	F1_Score	FNR
SVM	0.6667	0.5556	0.5100	0.5318	0.8580
KNN	0.7407	0.8120	0.4100	0.5449	0.7752
EB	0.6667	0.6210	0.3030	0.4073	0.8013
RF	0.7037	0.7500	0.3040	0.4326	0.7584
CNN	0.6837	0.6500	0.3920	0.4891	0.7832
RNN1	0.7237	0.7730	0.3710	0.5014	0.7657
RNN2	0.7342	0.7420	0.4200	0.5364	0.7983
ECP	**0.8148**	**0.8571**	**0.6000**	**0.7059 (*k*** = **14)**	**0.7321**

**Figure 1:**
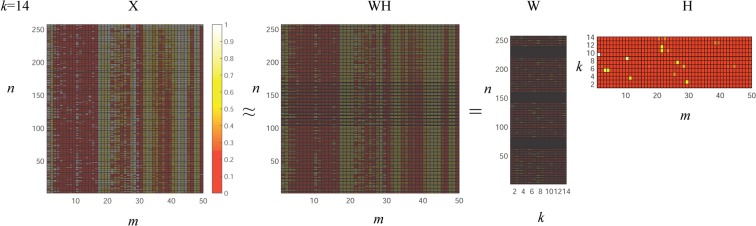
The base and coefficient matrices obtained after NMF.

**Figure 2:**
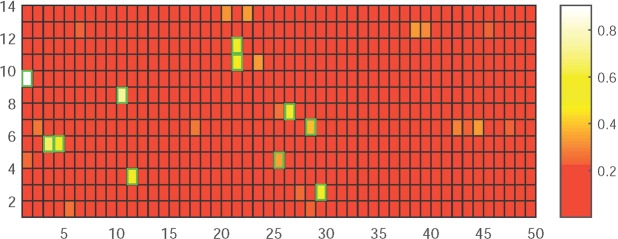
Heat map of the weight matrix between the attributes and the extracted features.

Next, we analyze the multidimensional features of the original dataset. In this paper, we input *m* attributes and *n* samples, where *X*_*ij*_ corresponds to the *j*^*th*^ attribute eigenvalue of the *i*^*th*^ sample. Here, *k* is a hypothetical number of important features in the NMF, which is generally less than the number of attributes. After NMF decomposition, *W*_*ik*_ corresponds to the correlation probability of the *i*^*th*^ sample and the *k*^*th*^ important feature, and *H*_*kj*_ corresponds to the probabilistic correlation of the *j*^*th*^ attribute and the *k*^*th*^ important feature. The result of the NMF is as follows:

We can see from Fig. 1 that after the nonnegative matrix decomposition the matrix retains the content of both the original matrix and the original *X* matrix in the dimensionally reduced *W* matrix. Finally, we construct a heat map of the properties of the *H* matrix in the nonnegative matrix decomposition and the *k* important features. We use the green block diagram to identify the most important attributes and features among all 50 attributes and the extracted 14 important features, as shown in Fig. 2 below:

As Fig. 2 shows, factors such as gender, smoking history, drinking history, hypertension, diabetes, whether early cancer is present, whether multiple cancers are present, whether lobes are used, and whether thermal biopsy forceps are used all have a greater impact on the characteristics of the extracted features after dimensionality reduction. For example, in patients with early stage cancer, the polyps are relatively large; thus, they are easily detected by thermal biopsy forceps. The use of thermal biopsy forceps is correlated with the detection of early cancer.

**Figure 3:**
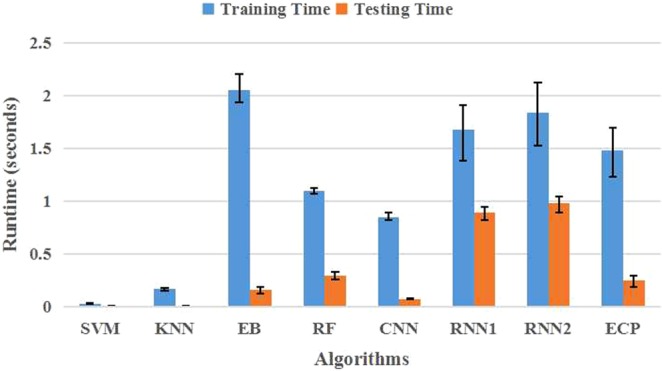
The runtime of different algorithms. The results are obtained by averaging over 10 realizations. The error bars show the standard deviations.

To further compare the computational efficiency of these methods, the processing speed of each method was recorded and listed in the figure shown in Fig. 3. As shown in the figure, by averaging the runtime during the training and testing procedure over 10 realizations, we find that our proposed method ECP have a medium runtime compared with the other Deep Learning methods. The RNN1 and RNN2 methods were less efficient than the other methods, especially for the testing runtime.

In summary, this model can help to improve the efficiency of early cancer diagnosis. In contrast to conventional deep learning techniques that focus on image processing, which is usually highly time consuming, our algorithm uses a deep learning technique to analyze patient case reports. This approach not only reduces the spatial complexity of the sample but also achieves better prediction results. In addition, our model suggests that several items in the examinations, such as “smoking history”, “drinking history”, “hypertension”, and “diabetes”, are highly correlated with the occurrence of cancer.

## Discussion

### Case source

The data are a collection of clinical patient records with intraepithelial neoplasia revealed by total colonoscopies performed at the endoscopy center of the First Affiliated Hospital of Nanjing University of Traditional Chinese Medicine (Jiangsu Provincial Hospital of Traditional Chinese Medicine) from February 2014 to February 2016. All the patients provided informed consent as follows: Before the study, the purported benefits and risks of the study, the endoscopic minimally invasive treatment method, its effectiveness, safety, and so on were explained to the patient, and if necessary, to family members; then, the patient or family signed a surgical consent form along with the informed consent form, and the hospital and patient each hold one copy of the forms. For hospitalized patients, doctors have the relevant healthcare records. The observations contained in these records are as follows: (1) patient name, gender, date of birth, birth place, contact information, contact address, height, weight, past history, and family history; (2) number of adenomas, lesions, size, shape classification, glandular opening pit pattern classification, lobulation, treatment, postoperative pathology, etc.

#### Inclusion criteria

(1) The tumor is located in the colorectal. (2) There is no contraindication to general anesthesia and surgery, and surgery is performed. (3) The pathology diagnosis after endoscopic surgery is intraepithelial neoplasia. (4) The clinical statistics are complete. (5) Informed consent and voluntary participation in clinical research are provided.

#### Exclusion criteria

(1) Cases with clinical signs of colorectal intraepithelial neoplasia who showed metastasis. (2) Patients with progressive CRC. (3) Patients who had taken anticoagulant drugs such as aspirin and clopidogrel within a week or patients with severe coagulopathy. (4) Cases with severe cardiopulmonary dysfunction or patients at risk from other endoscopic treatments. (5) Pregnant or lactating women. (6) Partially or completely restricted consciousness and behavior as determined by ability.

**Research case termination**: The research will be terminated under the following conditions: (1) Patients who have accidents during the treatment process or who need to undergo surgical treatment. (2) Patients who need additional surgery or other treatment methods after the endoscopic minimally invasive treatment. (3) Patients who have medical disputes.

**Figure 4:**
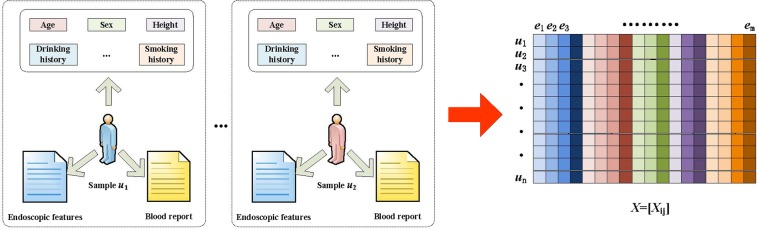
Mathematical problem modeling, converting nonstructural data into structural data.

To design the algorithm, we use the following data structure to store the sample data. We define *A* = {*u*_*i*_,*e*_*j*_,*x*_*ij*_} as the patient’s sample data, where *u*_*i*_ is patient *i* from the samples, *e*_*j*_ is attribute *j* in the sample, and *x*_*ij*_ is the value of attribute *j* from sample *i*. Assuming that the sample data include *n* patients and *m* attributes, the sample data constitute an n*m matrix *X* = [*x*_*ij*_]. Figure 4 shows an example of a sample dataset.

The early cancer prediction method attempts to assign a tag *y*_*i*_ ∈ {0, 1} to each new sample vector *x*_*i*_ = {*x*_*i*1_, *x*_*i*2_, ..., *x*_*im*_} to be predicted. For the case sample, a 1 indicates that the prediction is early cancer, while a 0 indicates cases not predicted as early cancer. To test the accuracy of the algorithm, a sample dataset with known tags must be divided into a training set and a test set. Only the information in the test set is allowed to be used when calculating the labels for the predicted samples. Obviously, $X={X}^{train}\cup {X}^{test}$, and ${X}^{train}\cap {X}^{test}=\O$. Each of the experimental results is averaged over 100 runs with randomly divided data where 10% of the entire dataset is used as a test set, and the other 90% of the data is used as a training set.

### Algorithm evaluation

After designing the prediction algorithm, we need to evaluate its outcome. Currently, the commonly used indicators for measuring the accuracy of such algorithms are accuracy, precision, recall, F1_Score and FNR. We used a 2 × 2 confusion matrix to describe the four possible prediction outcomes:

- A true positive (TP) means that the predictive model correctly predicted a positive category sample as a positive category.
- A true negative (TN) means that the predictive model correctly predicted a negative category sample as a negative category.
- A false positive (FP) means that the predictive model incorrectly predicted a negative category sample as a positive category.
- A false negative (FN) means that the predictive model incorrectly predicted a positive category sample as a negative category.

1. Accuracy is the ratio of the model’s prediction of correct results to the total results, as defined below:
  1 $$Accuracy=\frac{TP+TN}{TP+TN+FP+FN}$$
2. Precision is the proportion of positive categories in samples that are identified as positive categories, as defined below:
  2 $$Precision=\frac{TP}{TP+FP}$$
3. Recall is the proportion of samples that are correctly identified as positive categories among all the positive category samples, as defined below:
  3 $$Recall=\frac{TP}{TP+FN}$$
4. F1_Score combines the results of precision and recall; it is the weighted average of precision and recall. When the F1_Score is high, the test method can be regarded as effective. The F1_Score is defined as follows:
  4 $$F1\_Score=\frac{2\ast Precision\ast Recall}{Precision+Recall}=\frac{2\ast TP}{2\ast TP+FP+FN}$$
5. The false negative rate (FNR) is the number of patients who actually had cancer but were not recognized as having cancer, divided by the number of actual cancer patients: The definition is as follows:

5 $$FNR=\frac{FN}{TP+FN}$$

Table 2 describes the model predictions for all four possible outcomes as an example:

**Table 2:** An example used to describe different metrics, the actual values include 100 samples of cancer (positive category) or noncancer (negative category).

True Positives (TP):	False Positives (FP):
Actual: Cancer	Actual: No Cancer
Predicted: Cancer	Predicted: Cancer
Number of TP cases: 1	Number of FP cases: 1
False Negatives (FN):	True Negatives (TN):
Actual: Cancer	Actual: No Cancer
Predicted: No Cancer	Predicted: No Cancer
Number of FN cases: 8	Number of TN cases: 90

Here, the accuracy is 0.91, which means that 91% (91 out of 100 samples) are correct. This might seem to be a good result; however, of the nine early cancer samples, only one of the nine cases was correctly identified as cancer. This result is not satisfactory because 8 of the 9 cancer cases were not diagnosed correctly. Therefore, when we use an imbalanced dataset (where a significant difference exists between the number of positive and negative category labels), accuracy alone does not reflect the true situation.

Precision is the ratio of the positive category in the sample identified as a positive category. In this example, we calculate that the precision equals 0.5; meanwhile, we find that the recall equals 0.11, the F1-Score equals 0.18, and the FNR equals 0.88. These two indicators show that the toy model used above performs rather poorly. Therefore, we can see that the F1-score and FNR metrics can be used to effectively evaluate the prediction model when the data samples are not balanced.

### The optimal choice of dimension

**Figure 5:**
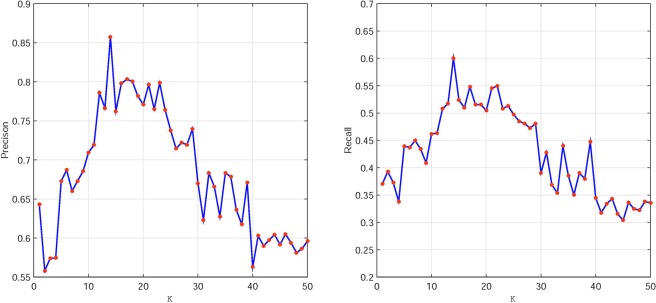
The variation tendencies of precision and recall with different *k*.

In the experiments, *k* is the dimension of the matrix attribute after dimensionality reduction using nonnegative matrices, that is, the number of important features to be extracted. Because the dimension of the original dataset matrix is 50, we gradually increase the dimension (*k*) of the nonnegative matrix after dimensionality reduction from 1 to 50. We find that the algorithm achieves its best performance when *k* = 14. Simultaneously, we also show the evaluation metrics of our method as *k* changes from 1 to 50 in the experiment. We calculate the variation of two evaluation metrics (precision and recall) with different dimensions of the input features. The results are shown in Fig. 5.

### The advantages of ECP

Compared with the other algorithms, negative values are not considered in dimensionality reduction because a negative value in the sample data matrix has no real-world meaning in early cancer prediction. For example, during prediction, if a negative value appears in the sample, the characteristics of the sample data will never be selected during the feature extraction process. However, this situation may not be correct because the feature may become significant and play a key role in the future. Our model not only reduces the dimensionality of the matrix but also removes redundant data, making the decomposed result more interpretable in practice.

In addition, because our sample data are small, an SVM can easily find a linear relationship between the data and the features for small and medium sample sizes, thereby avoiding the use of a neural network structure and its attendant local minimal value problems. The method is highly interpretable and can be used to solve high-dimensional problems. In addition, the algorithmic time complexity of linear SVM is significantly lower.

## Methods

### Ethics approval and consent to participate

The present study was approved by The Ethics Committee of the Affiliated Huaian Hospital of Xuzhou Medical University. All patients provided written informed consent before participating, and all the methods were conducted in accordance with the relevant guidelines and regulations.

### Deep learning framework of early cancer prediction algorithm based on nonnegative matrix

Based on the iterative method for NMF computing, we present an algorithm for early cancer prediction based on NMF, named ECP. The framework of our algorithm is shown below.

**Algorithm:**
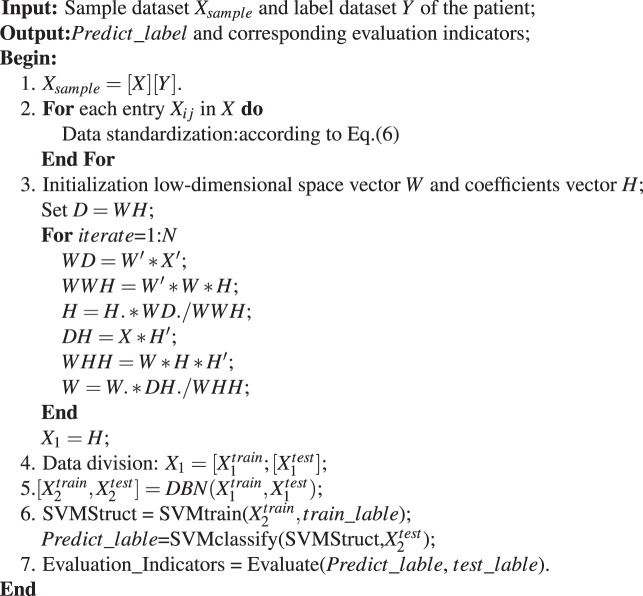
EarlyCancer_Prediction(ECP Algorithm).

### Detailed algorithm steps

#### Data standardization

In the early cancer prediction algorithm, we need to process multidimensional patient sample data. First, we need to standardize the sample data. Data standardization is based on the column of the feature matrix for data processing. The Z-score standardization method, which standardizes the attributes of each dimension of the sample, is widely used in many deep learning algorithms. This method uses the mean and standard deviation of the data to standardize the data so that the processed data conform to a standard normal distribution, i.e., with a mean of 0 and a standard deviation of 1. After normalizing the data, the error caused by the different feature characteristics of each attribute cancel out, and the standardization is a linear transformation, which involves converting a certain characteristic attribute in the sample data according to its proportional compression. Data standardization can improve the performance of the data without having to change the numerical ordering of the original data. The specific standardized function is as follows:

6 $$X^{\prime} =\frac{X-\mu }{\sigma },$$

where *μ* is the mean of the attribute data for each column of the sample, and *σ* is the standard deviation of the attribute data for each column of the sample.

#### Eigenvalue extraction

To address the high dimensionality and redundancy characteristic of the sample data, we need to effectively reduce the dimensionality of the original network’s sample matrix to remove redundant attributes. For example, certain factors (such as name, gender and age) exist in the dataset that we can reasonably believe would not provide a positive contribution to the prediction algorithm model. Therefore, we can use a method to remove these redundant attributes and improve the final accuracy of the prediction algorithm.

Although some matrix dimensionality reduction methods have been used in cancer prediction, they do not consider the actual situation in clinical medicine. For example, during sample testing, blood samples will have only nonnegative values. However, common dimensionality reduction methods produce negative values in the data matrix of the sample after dimensional reduction, which is a nonphysical result. Meanwhile, because each feature is evaluated independently, such screening methods may fail to capture all the highly discriminative feature subsets, each of which is composed of less discriminative features.

**Figure 6:**
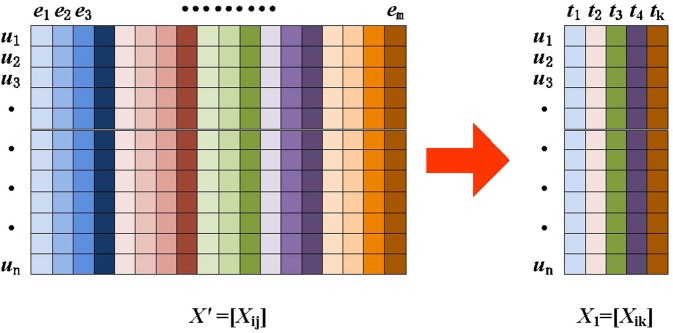
Illustration of NMF, which extracts k-dimensional representative features from the m-dimensional features (k < m).

Therefore, at the beginning of the algorithm, we use the NMF method as the matrix decomposition technique to reduce the dimensionality of the sample dataset and then approximate the original matrix using the decomposed matrix and the weight matrix to reduce the time and space complexities of the algorithm. In this paper, NMF is applied to the prediction of early cancer diseases as shown in Fig. 6. The correlation between the different types of matrices is reconstructed by projecting a high-dimensional vector space into a low-dimensional vector space. The algorithm reduces the storage space of the data while maintaining a low time complexity and can effectively improve the prediction performance.

The traditional dimensionality reduction method is used to statistically analyze only the sample attributes and data, without considering other information. NMF is different; it can often represent nonlocal correlations to obtain better prediction results. We can regard the sample matrix as a nonnegative feature matrix, where each row represents the eigenvector of a sample. The goal of NMF is to solve two nonnegative matrix factors *W* ∈ *P*_*n*_ * _*k*_ and *H* ∈ *P*_*k*_ * _*m*_, (*n* + *m*) * *k* < *nm* so that the product of the two approximates the matrix *X*:

7 $$X^{\prime} =WH,$$

where *k* represents the dimension of the low-dimensional space and *W* represents the low-dimensional space vector, called the base matrix. *H* denotes the coefficients of the vector product of the reconstructed original matrix, which is called a weight matrix. This decomposition problem is usually modeled as a Frobenius norm optimization problem:

8 $$\mathop{{\rm{\min }}}\limits_{u,v}{\Vert X^{\prime} -WH\Vert }_{F}^{2}\,s.t.W\ge 0,H\ge 0,$$

in which the constraints ensure that all the elements of the matrix *W*, *H* are nonnegative. In this paper, we replace the original matrix abs with a coefficient matrix that reduces the dimensions of the original matrix *X*′ to *k*. This operation not only reduces the required storage space but also retains the intrinsic information of the data insofar as possible after dimensionality reduction.

#### Data division

After the NMF process, we randomly divide the obtained *X*_1_ matrix into training data *X*_1_^*train*^ and test data *X*_1_^*test*^. The training data *X*_1_^*train*^ includes 90% of the data, and the remaining 10% constitute the test data *X*_1_^*test*^. It is important to note that we classify all records into two classes based on *Y* and randomly choose 90% of the records in each class to construct a training set to eliminate the imbalance effect of the sample data. The training data *X*_1_^*train*^ are used to train the DBN in the next step, after which the test data *X*_1_^*test*^ is used as the input to the DBN, which generates *X*_2_^*test*^ used as input to the final SVM.

#### The prediction model based on DBN

Given an insufficient number of data samples, some conventional machine learning methods do not achieve good results. For example, traditional neural networks generally have one or two hidden layers because, after the number of neurons becomes too large, there are too many hidden layers; consequently, the number of parameters in the model increases rapidly, and the model training time becomes increasingly long. Additionally, in traditional neural networks, as the number of layers increases, it becomes difficult to find the optimal solution by using random gradient descent, and the model can easily become trapped in locally optimal solutions. Gradient dispersion and gradient saturation are also prone to occur during backpropagation, resulting in unsatisfactory model results. Under increasing numbers of neural network layers, deep neural networks utilize many model parameters, which requires large amounts of labeled data during training because it is difficult to find the optimal solution when the training dataset is small. In general, deep neural networks are not a good fit for solving small-sample problems.

However, the DBN solves the problem of deep neural network optimization by adopting layer-by-layer training. Under layer-by-layer training, the entire network is given a reasonable initial weight; then, the optimal solution can be reached by simply refining the weights. Restricted Boltzmann machines (RBMs), which play an important role in the training process, are composed of visible layers and hidden layers. The visible layers accept input, and the hidden layers extract features. After training the RBM, the characteristics of the input data can be obtained, i.e., the invisible features of the input data are extracted.

Because of the above characteristics of RBM, DBN layer-by-layer training is effective. The hidden layer extraction feature makes the training data of subsequent levels more representative, and the problem of insufficient sample size can be solved by generating new data.

DBN performs model training in two main steps: Step 1: separately train each layer of the RBM network in an unsupervised manner and ensure that the maximum feature information is retained when the feature vector is mapped to different feature spaces. Step 2: Set the BP network as the last layer of the DBN, take the output feature vector of the RBM as its input feature vector, and train the entity relationship classifier in a supervised manner. Each layer of the RBM network can only ensure the weight value in its own layer. The feature vector mapping of this layer is optimal, whereas the feature vector mapping of the entire DBN is not optimal. Therefore, the back-propagation network also propagates the error information from top to bottom to each layer of the RBM and finally fine-tunes the DBN network. The RBM network training process can be regarded as the initialization of a deep BP network weight parameter, which allows the DBN to overcome the shortcomings of the BP network, where the latter falls readily into local optima and suffers from long training times due to the random initial weight parameters.

In this paper, we obtain the number of attribute features obtained by the nonnegative matrix decomposition as *K* = 14. After training the DBN, the last layer is our output feature. The dimension of the feature vector is the number of nodes in the last layer. The number of nodes is determined through parameter sensitivity experiments according to our data characteristics. We ultimately chose 4 as the number of nodes.

**Figure 7:**
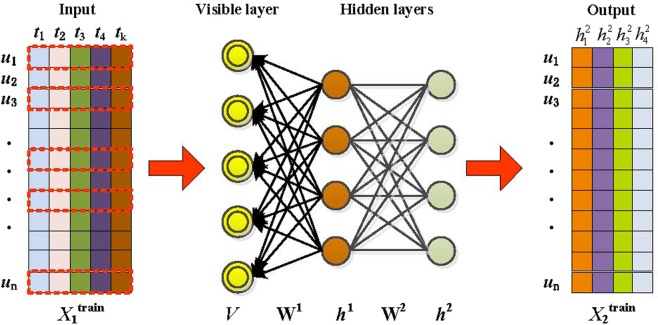
The architecture of the DBN-based feature extraction model.

In early cancer prediction, we take the attribute vector *V* of each case sample after dimensionality reduction as the input of the DBN, as shown in Fig. 7. In this training phase, the visible layer input vector *V* is passed to the hidden layer. Conversely, the input *V* of the visible layer is randomly selected to attempt to reconstruct the original input data. Finally, these new visible neuron activation units reconstruct the hidden layer activation unit forward to obtain *h*^1^ and *h*^2^. During the training, Gibbs sampling is performed to repeat the above process. The correlation difference between the activated units in the hidden layer and the input visible layer is used as the basis for the update of the weights *W*^1^ and *W*^2^.

The conditional probabilities under the input data and hidden layers are as follows:

9 $$P({h}_{j}^{1}|V)=g(\mathop{\sum }\limits_{i=1}^{|V|}\,{W}_{ij}^{1}{V}_{i}+{a}_{j}^{1})$$

10 $$P({h}_{k}^{2}|{h}^{1})=g(\mathop{\sum }\limits_{j=1}^{|{h}^{1}|}\,{W}_{jk}^{2}{h}_{j}^{1}+{a}_{k}^{2})$$

11 $$P({V}_{i}|{h}^{1})=g(\mathop{\sum }\limits_{j=1}^{|{h}^{1}|}\,{W}_{ijjk}^{1}{h}_{j}^{1}+{b}_{i}),$$

where *g* is the sigmoid function, which is defined as follows:

12 $$g(x)=\frac{1}{1+{e}^{-x}}$$

Here, *b*_*i*_ is the offset of the input layer, and *a*_*i*_ is the offset of the hidden layer. Through this step, we derive the user’s feature matrix *X*_2_ as the input to the next classification model.

#### Prediction model using an SVM

**Figure 8:**
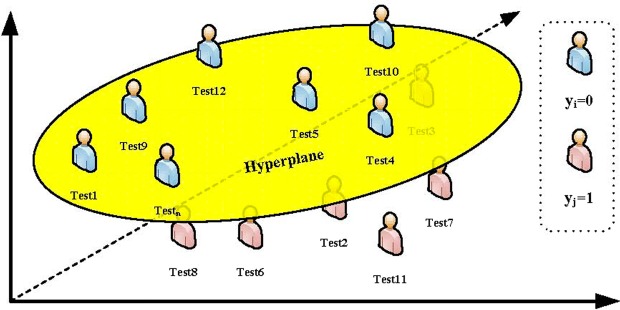
Illustration of the classification results using SVM.

In the final step of early cancer prediction, we transform the prediction problem into a classification problem. The basic idea of the classification algorithm is to find the dividing hyperplane in the feature space based on the training set *X*_2_^*train*^ that best separates the positive and negative samples. We map the original indivisible data to a new space and classify the converted data, as shown in Fig. 8.

We take the output feature matrix *X*_2_^*Train*^ of the DBN in the previous step as the training set of the classification algorithm, i.e., *X*_2_^*Train*^ = *x*_*i*_|*i* ∈ 1, ..., *n*, *y* ∈ 0, 1. Then, the linear SVM learns to obtain the separated hyperplane as follows:

13 $$w\cdot x+b=0$$

The corresponding classification decision function is

14 $$f(x)=sign(w\cdot x+b)$$

The two different classifications of the sample points closest to the separated hyperplane are called support vectors, and two long bands parallel to the separated hyperplane are formed. The distance from the hyperplane indicates the confidence of the classification; the greater the distance, the higher the confidence that the classification is correct. This value is easy to obtain by calculating the following:

15 $$margin=\frac{2}{||w||}$$

The SVM classification problem can be described as maximizing $\frac{2}{||w||}$ given that *y*_*i*_(*w*⋅*x*_*i*_ + *b*) ≥ 1, i.e.,

16 $$mi{n}_{w,b}=\frac{2}{||w||}\,s.t.{y}_{i}(w\cdot {x}_{i}+b)\ge 1$$

Next, by constructing a Lagrangian function and solving the partial derivative, an equivalent problem can be obtained:

17 $$\mathop{{\rm{\min }}}\limits_{a}\frac{1}{2}\mathop{\sum }\limits_{i=1}^{n}\,\mathop{\sum }\limits_{j=1}^{n}\,{a}_{i}{a}_{j}{y}_{i}{y}_{j}({x}_{i}\cdot {x}_{j})-\mathop{\sum }\limits_{i=1}^{n}\,{a}_{i}\,s.\,t.\mathop{\sum }\limits_{i=1}^{n}\,{a}_{i}{y}_{i}=0,$$

where *a*_*i*_ ≥ 0 is the Lagrange multiplier. In the problem of early cancer prediction, we take the result *X*_2_ of the DBN output in the previous step as the input of the SVM classification algorithm. Next, we obtain the training model. Finally, we obtain the prediction result (Predict_label) corresponding to the test set (test_data).

## References

[1] Chen W (2016). Cancer statistics in china, 2015. CA: a cancer journal for clinicians.

[2] Society, A. C. Cancer facts and figures: 2017. *CA: a cancer journal for clinicians* (2017).

[3] Courtney RJ (2013). A population-based cross-sectional study of colorectal cancer screening practices of first-degree relatives of colorectal cancer patients. BMC cancer.

[4] Carter JV (2016). A highly predictive model for diagnosis of colorectal neoplasms using plasma microrna: improving specificity and sensitivity. Annals surgery.

[5] Yuan P, Gu J (2017). Meta-analysis of the compliance of colorectal cancer screening in china, 2006–2015. China Cancer.

[6] Chen X, Tan X, Li L (2013). Health problem and occupational stress among chinese doctors. Chin. Medicine.

[7] He AJ, Qian J (2016). Explaining medical disputes in chinese public hospitals: the doctor–patient relationship and its implications for health policy reforms. Heal. Econ. Policy Law.

[8] Wu D, Wang Y, Lam KF, Hesketh T (2014). Health system reforms, violence against doctors and job satisfaction in the medical profession: a cross-sectional survey in zhejiang province, eastern china. BMJ open.

[9] Wu H, Ge CX, Sun W, Wang JN, Wang L (2011). Depressive symptoms and occupational stress among chinese female nurses: the mediating effects of social support and rational coping. Res. nursing & health.

[10] Jingang A (2013). Which future for doctors in china?. The Lancet.

[11] Cammà C (2000). Preoperative radiotherapy for resectable rectal cancer: a meta-analysis. Jama.

[12] Guimerà R, Sales-Pardo M (2009). Missing and spurious interactions and the reconstruction of complex networks. Proc. Natl. Acad. Sci..

[13] Esteva A (2017). Dermatologist-level classification of skin cancer with deep neural networks. Nat..

[14] Kourou K, Exarchos TP, Exarchos KP, Karamouzis MV, Fotiadis DI (2015). Machine learning applications in cancer prognosis and prediction. Comput. structural biotechnology journal.

[15] Wang L, Wang Y, Chang Q (2016). Feature selection methods for big data bioinformatics: A survey from the search perspective. Methods.

[16] Kleftogiannis D, Theofilatos K, Likothanassis S, Mavroudi S (2015). Yamipred: A novel evolutionary method for predicting pre-mirnas and selecting relevant features. IEEE/ACM transactions on computational biology bioinformatics.

[17] Duan K-B, Rajapakse JC, Wang H, Azuaje F (2005). Multiple svm-rfe for gene selection in cancer classification with expression data. IEEE transactions on nanobioscience.

[18] Zhong J, Wang J, Peng W, Zhang Z, Li M (2015). A feature selection method for prediction essential protein. Tsinghua Sci. Technol..

[19] Fong S, Deb S, Yang X-S, Li J (2014). Feature selection in life science classification: metaheuristic swarm search. IT Prof..

[20] Mohapatra P, Chakravarty S, Dash P (2016). Microarray medical data classification using kernel ridge regression and modified cat swarm optimization based gene selection system. Swarm Evol. Comput..

[21] Metsis V, Makedon F, Shen D, Huang H (2014). Dna copy number selection using robust structured sparsity-inducing norms. IEEE/ACM Transactions on Comput. Biol. Bioinforma. (TCBB).

[22] Boareto M, Cesar J, Leite VB, Caticha N (2014). Supervised variational relevance learning, an analytic geometric feature selection with applications to omic datasets. IEEE/ACM transactions on computational biology bioinformatics.

[23] Bennasar M, Hicks Y, Setchi R (2015). Feature selection using joint mutual information maximisation. Expert. Syst. with Appl..

[24] Xu X, Li A, Wang M (2015). Prediction of human disease-associated phosphorylation sites with combined feature selection approach and support vector machine. IET systems biology.

[25] Das, L., Das, J. & Nanda, S. Advanced protein coding region prediction applying robust svd algorithm. In 2017 2^nd^*International Conference on Man and Machine Interfacing (MAMI)*, 1–6 (IEEE, 2017).

[26] Cobos C (2013). A hybrid system of pedagogical pattern recommendations based on singular value decomposition and variable data attributes. Inf. Process. & Manag..

[27] Qureshi, N. A. *et al*. Application of principal component analysis (pca) to medical data. *Indian J. Sci. Technol*. **10** (2017).

[28] Hyvärinen, A., Karhunen, J. & Oja, E. *Independent component analysis*, vol. 46 (John Wiley & Sons, 2004).

[29] Funk, S. *Netflix update: Try this at home* (2006).

[30] Koren Y (2010). Factor in the neighbors: Scalable and accurate collaborative filtering. ACM Transactions on Knowl. Discov. from Data (TKDD).

[31] Lee DD, Seung HS (1999). Learning the parts of objects by non-negative matrix factorization. Nat..

[32] Lee, D. D. & Seung, H. S. Algorithms for non-negative matrix factorization. In *Advances in neural information processing systems*, 556–562 (2001).

[33] Wang, F., Zhang, P. & Dudley, J. Healthcare data mining with matrix models. In *Proceedings of the 22nd ACM SIGKDD International Conference on Knowledge Discovery and Data Mining*, 2137–2138 (ACM, 2016).

[34] Sandler R, Lindenbaum M (2011). Nonnegative matrix factorization with earth mover’s distance metric for image analysis. IEEE Transactions on Pattern Analysis Mach. Intell..

[35] Nikitidis S, Tefas A, Nikolaidis N, Pitas I (2012). Subclass discriminant nonnegative matrix factorization for facial image analysis. Pattern Recognit..

[36] Leng, C. *et al*. Total variation constrained graph regularized nmf for medical image registration. In *2016 IEEE 12th Image, Video, and Multidimensional Signal Processing Workshop (IVMSP)*, 1–5 (IEEE, 2016).

[37] Reda, I. *et al*. A new nmf-autoencoder based cad system for early diagnosis of prostate cancer. In *2016 IEEE 13*^*th*^*International Symposium on Biomedical Imaging (ISBI)*, 1237–1240 (IEEE, 2016).

[38] Li Y, Ngom A (2013). The non-negative matrix factorization toolbox for biological data mining. Source code for biology medicine.

[39] Xiao Y, Wu J, Lin Z, Zhao X (2018). A deep learning-based multi-model ensemble method for cancer prediction. Comput. methods programs biomedicine.

[40] Danaee, P., Ghaeini, R. & Hendrix, D. A. A deep learning approach for cancer detection and relevant gene identification. In *PACIFIC SYMPOSIUM ON BIOCOMPUTING* 2017, 219–229 (World Scientific, 2017). 10.1142/9789813207813_0022 PMC5177447 27896977

[41] Bychkov D (2018). Deep learning based tissue analysis predicts outcome in colorectal cancer. Sci. reports.

[42] Cruz-Roa, A. A., Ovalle, J. E. A., Madabhushi, A. & Osorio, F. A. G. A deep learning architecture for image representation, visual interpretability and automated basal-cell carcinoma cancer detection. In *International Conference on Medical Image Computing and Computer-Assisted Intervention*, 403–410 (Springer, 2013). 10.1007/978-3-642-40763-5_50 24579166

[43] Coudray N (2018). Classification and mutation prediction from non–small cell lung cancer histopathology images using deep learning. Nat. medicine.

[44] Mamoshina P, Vieira A, Putin E, Zhavoronkov A (2016). Applications of deep learning in biomedicine. Mol. pharmaceutics.

[45] Burke HB (1997). Artificial neural networks improve the accuracy of cancer survival prediction. Cancer.

